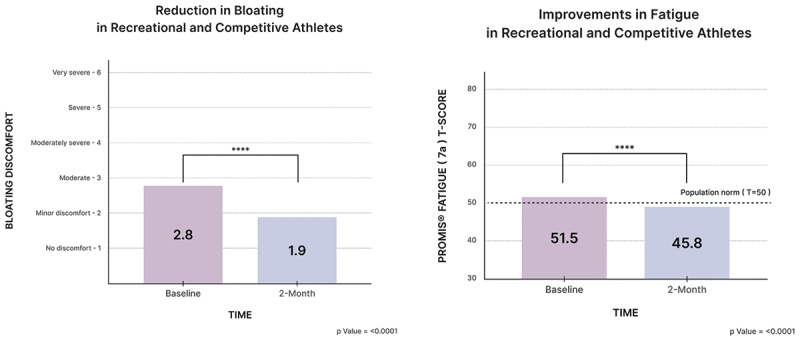# A daily three-strain probiotic improves fatigue levels and reduces bloating in recreational and competitive athletes

**DOI:** 10.1080/15502783.2025.2550151

**Published:** 2025-09-11

**Authors:** Tara Karr, Marin Thompson, Renee Korczak, Adam Perlman

**Affiliations:** Pendulum Therapeutics Inc., San Francisco, CA, USA

**Keywords:** Probiotic supplement, athletic performance, bloating, fatigue

## Abstract

**Background:**

Probiotics nourish the community of microorganisms that are part of the larger gut microbiome. As an emerging area of research, few studies have explored the effects of specific probiotic strains on athlete health and performance. The primary aim of this study was to evaluate the effects of a three-strain probiotic consumed for two months in athletes on measures of fatigue and markers of Gastrointestinal Health (GI).

**Methods:**

A study on athletic consumers was conducted to assess the effect of three-strain Pendulum Probiotic, taken daily with food, for two consecutive months on self-reported levels of fatigue and bloating. The product contains 500 million active fluorescent units (AFU) of probiotic bacteria, consisting of *Clostridium butyricum* WB-STR-0006, *Akkermansia muciniphila* WB-STR-0001, *Bifidobacterium infants*, and 211 mg of prebiotic inulin. Participants were a mix of recreationally active through elite-level athletes (*n* = 330; 263 F/67 M) who were categorized into 4 athletic tiers, based on activity and performance level^1^. Participants answered surveys at baseline and at 2 months. Fatigue was assessed using the validated PROMIS (7a) Fatigue short form^2^ to generate t-scores, with lower scores indicating better function. Bloating was assessed using a modified GI ranking severity score.

**Results:**

For bloating, significant improvements were observed for athletes from baseline to two months (2.8 vs. 1.8 baseline vs. 2 months, MD = −0.96, *p* < 0.00001). For fatigue, participants experienced a statistically and clinically significant reduction in fatigue over two months, with PROMIS (7a) Fatigue scores improving from 51.5 to 45.8 (*p* = 6.652 e-31). This 5.7 point reduction and the fact that final scores fell below the population norm (*T* = 50), indicates meaningful improvements in reduced fatigue.

**Conclusions:**

Daily supplementation of three three-strain Pendulum Probiotic was associated with significant improvements in self-rated markers of fatigue and bloating. Previous research suggests these benefits are likely driven by the mechanistic ability of the three probiotic strains to strengthen gut barrier integrity, enhance microbial diversity and increase short-chain fatty acid production. These mechanisms may help improve digestion, and sustain energy metabolism. These findings suggest that a three-strain Pendulum Probiotic may help support performance by improving unwanted bloating and fatigue.

**Figure:**